# Microbiome Shifts in Bladder Cancer: A Narrative Review of Urobiome Composition, Progression, and Therapeutic Impact

**DOI:** 10.3390/medicina61081401

**Published:** 2025-08-01

**Authors:** Raul-Dumitru Gherasim, Călin Chibelean, Daniel Porav-Hodade, Ciprian Todea-Moga, Sabin-Octavian Tătaru, Tibor-Lorand Reman, Arpad-Oliver Vida, Maria-Veronica Ghirca, Matteo Ferro, Orsolya Katalyn Ilona Martha

**Affiliations:** 1 Department of Urology, George Emil Palade University of Medicine, Pharmacy, Science, and Technology of Târgu Mureș, 540139 Târgu Mureș, Romania; raul-dumitru.gherasim@umfst.ro (R.-D.G.); daniel.porav-hodade@umfst.ro (D.P.-H.); ciprian.todea@umfst.ro (C.T.-M.); tibor.reman@umfst.ro (T.-L.R.); arpad.vida@umfst.ro (A.-O.V.); maria.ghirca@umfst.ro (M.-V.G.); orsolya.martha@umfst.ro (O.K.I.M.); 2 Department of Urology, Târgu Mureș Clinical Hospital, P-ța Bernády György, Nr. 6, 540072 Tîrgu-Mureș, Romania; 3 Institution Organizing University Doctoral Studies, George Emil Palade University of Medicine, Pharmacy, Science and Technology, 540142 Targu Mures, Romania; sabin.tataru@gmail.com; 4 Unit of Urology, Department of Health Science, Faculty of Medicine, University of Milan, ASST Santi Paolo and Carlo, 20122 Milan, Italy; matteo.ferro@unimi.it

**Keywords:** urinary microbiome, bladder cancer, microbial biomarkers, intravesical therapy, public health burden

## Abstract

*Background/Objectives*: Bladder cancer is a common malignancy with a high rate of recurrence and progression. Recent studies have identified that the urinary microbiome can be a key factor in tumor pathogenesis, progression, and outcomes. This narrative review is designed to summarize current evidence regarding the urobiome and explore its diagnostic and therapeutic potential. *Methods:* Studies between 2019 and 2024 were identified through the PubMed/MEDLINE and Google Scholar databases. Case reports and non-English-language articles were excluded. *Results:* The main findings revealed that specific bacteria, viruses, and taxa are linked to bladder cancer presence, progression, and response to immunotherapy treatment. Urinary microbiota differ by tumor type, sex, smoking status, and occupational exposure to toxins. *Conclusions:* Urinary microbiome and certain types of viruses present in urine may serve as promising tools to enhance bladder cancer diagnosis and predict treatment response. However, larger longitudinal studies are needed to confirm and establish these findings. Furthermore, integration of the urinary microbiome in clinical practice and public health strategies may reduce disease-related burden.

## 1. Introduction

Bladder cancer is a common malignancy worldwide, with approximately 573,000 new cases and 213,000 deaths reported in 2020. Its incidence is three to four times higher in men than in women [1]. Well-established risk factors include tobacco smoking and occupational exposure to carcinogens, yet the disease’s pathophysiology remains only partially understood [2]. For decades, the bladder was thought to be a sterile environment, but this dogma has been overturned by modern DNA-based techniques that reveal a resident urinary microbiome [3].

Metagenomic analyses have shown that the urinary tract harbors over 100 different microbial species across more than 50 genera (with Lactobacillus, Gardnerella, and Streptococcus among the most abundant) [4]. This discovery has prompted investigations into how these microbial communities (sometimes termed the “urobiome”) might influence urinary tract health and disease.

Urinary microbiome dysbiosis (imbalance) has been linked to various benign urological disorders, including interstitial cystitis, bladder dysfunction, and urothelial carcinoma [5].

Emerging evidence also suggests a connection between the urobiome and bladder cancer risk [6].

The interest in how microbial imbalances contribute to bladder cancer constantly grows. The growing interest in the urinary microbiome is supported by findings from related fields, such as the well-established role of the gut microbiome in colorectal cancer [7], and by the emerging hypothesis that microbially induced inflammation may contribute to bladder tumorigenesis. Although chronic inflammation is a well-established driver in various malignancies [8], the specific contribution of the urinary microbiome to bladder cancer pathogenesis remains incompletely understood, yet highly promising.

Several studies have hypothesized that certain urinary microbial communities may facilitate tumor development by promoting persistent inflammation or by generating carcinogenic metabolites [9]. However, findings across microbiome studies in bladder cancer have been inconsistent—while some analyses reveal significant differences in microbial composition and diversity between patients with bladder cancer and healthy controls, others report no notable disparities. These discrepancies may be attributed to differences in study design, sequencing techniques, and heterogeneity in patient populations, highlighting the need for standardized, large-scale investigations to clarify the urinary microbiome’s role in bladder carcinogenesis.

The temporal relationship between microbial alterations and bladder cancer remains a subject of debate, with uncertainty as to whether these changes precede tumor development or arise as a consequence of the disease. Although a clear association between the urobiome and bladder cancer has been demonstrated, the underlying causal mechanisms are yet to be fully elucidated. In light of its clinical potential, ongoing research is increasingly focused on exploring how the urinary microbiome may be harnessed to improve diagnostic, prognostic, and therapeutic outcomes in bladder cancer.

Advancing our understanding of this relationship could pave the way for the development of non-invasive screening tools, novel biomarkers, and preventive strategies aimed at reducing the public health burden associated with bladder cancer—underscoring the significant translational potential of this research domain.

Bladder cancer is costly to monitor and treat over time due to its high recurrence and progression rates. Improved early detection may lead to lower healthcare costs, reduce patients’ burden, and even improve the outcomes of this pathology. Additionally, modifiable factors such as the microbiome could open up new prevention methods that go beyond the traditional smoking cessation.

Another known fact is that around 70% of the patients with non-muscle invasive bladder cancer (NMIBC) experience tumor recurrence. Preventing recurrence is one of the most important methods we can offer our patients [10]. Our standard of care, according to the European Association of Urology (EAU), includes trans-urethral resection of bladder tumor (TURBT) followed by intravesical chemotherapy. A close follow-up, including regular cystoscopies, is required afterwards. The potential effects of these therapies on urinary microbiome (UB) dysbiosis warrant consideration [11]. Moreover, the interplay between the microbiome and cancer therapy is a cutting-edge topic. There is growing interest in whether the urinary microbiome can modulate responses to immunotherapy or intravesical therapy, analogous to findings in other cancers, where gut microbiota composition influences the efficacy of immune checkpoint inhibitors.

We can define UB as the collection of microbial genomes and live microorganisms present in urine. It has generated significant interest due to its potential role in bladder cancer development and immune system modulation. At present, urine is being re-evaluated through advanced molecular techniques. Emerging evidence suggests that it harbors a distinct microbial community [12]. There are multiple ongoing studies aimed at better characterizing the UB in both benign and malignant urinary tract conditions. It is important to establish sex-based differences regarding UB because it may explain the higher incidence of bladder cancer among men, with meaningful public health implications [13]. Similarly to the gut or skin, the urinary system is an interface between the immune system and the external environment. As mentioned before, UB is exposed to carcinogens such as tobacco smoke and chemicals, which lead to bladder cancer. Tumor mutational burden (TMB) is at its highest levels in this pathology [14] and can be associated with response to certain checkpoint inhibitors (anti-PD-1, anti-CTLA-4). These findings emphasize the influence the UB has on cancer risk, treatment outcomes, and healthcare resource allocation.

Given the potential to serve as a biomarker and therapeutic response marker, UB could become a precious tool in prevention, early detection, and follow-up in bladder cancer patients. If integrated into population health strategies, it could facilitate risk stratification and contribute to targeted and cost-effective care of NMIBC [15].

The aim of this study is to compile current knowledge on the urinary microbiome (UB) in bladder cancer—specifically non-muscle-invasive bladder cancer (NMIBC)—and to evaluate its implications for diagnosis, therapeutic strategies, public health, and sex-based cancer disparities

## 2. Materials and Methods

We conducted a comprehensive literature search using the PubMed/MEDLINE and Google Scholar databases. The search was limited to articles published between January 2019 and April 2024, focusing on studies relevant to the urinary microbiome and bladder cancer. The following keywords were used: “urinary bladder microbiome” and “bladder cancer”. Only articles published in English were considered. We excluded all case reports, conference abstracts, and non-peer-reviewed publications. After screening 115 articles, a total of 31 were relevant and included in this narrative review. The aim of this study was to synthesize current knowledge on UB in bladder cancer, specifically NMIBC, and to assess its implications in diagnosis, therapeutic measures, and also to explore its relevance in public health and sex-based disparities in cancer incidence.

## 3. Results

### 3.1. Microbiome in Male Patients

The urinary microbiome in healthy male individuals and its composition remain incompletely characterized. It is known that the urinary microbiome (UB) can differ with age due to benign prostatic hyperplasia (BPH), lower urinary tract symptoms (LUTS), and bladder outlet obstruction (BOO). Nelson et al. first applied 16S rRNA sequencing to urine samples from asymptomatic patients in a sexually transmitted disorders (STD) clinic, identifying 72 bacterial taxa, predominantly Lactobacillus, Streptococcus, and Sneathia. [16]. More studies on healthy adolescents confirmed similar microbial compositions, further supporting the presence of a specific urinary microbiota [17].

Despite age-related urological pathologies, the UB of elderly male patients remains under-investigated. Bajic et al. addressed this issue by analyzing the urine of male patients aged between 40 and 85 years [18]. The study concluded that the International Prostate Symptom Score (IPSS) was positively associated with bacterial abundance, raising concerns about dysbiosis and the severity of LUTS.

To conclude all the findings, a “normal” male urinary microbiome is not sterile, but contains a consistent set of commensal and low-virulence taxa, especially Lactobacillus, Corynebacterium, and Streptococcus. With increasing age, the microbiome appears to become more diverse and complex, integrating anaerobic and less common genera. These changes may be physiological or environmentally driven; studying these changes is crucial when we analyze dysbiosis in conditions such as bladder cancer [19].

The presented studies raise an important question: Can UB help in the therapeutic response of these patients? From a public health standpoint, understanding changes over time of UB and dysbiosis may offer new strategies for treatment and personalized management.

### 3.2. Microbiome in Female Patients

Roth et al. [17] also collected midstream urine samples from healthy females, with negative urine culture (defined as fewer than 10^5^ CFU/mL), using 16S rRNA gene sequencing to further analyze the data. Among the most commonly discovered microorganisms, Prevotella, Lactobacillus, and Gardnerella species were detected.

Similarly, Wolfe et al. used 16S rRNA sequencing to analyze urine from asymptomatic patients, revealing a diverse urinary microbiota dominated by species such as Lactobacillus, Gardnerella, Prevotella, Acinobaculum, Aerococcus, and Streptococcus [20].

In a prospective single-center cohort study, Price et al. used EQUC and 16S rRNA sequencing on urine from eight healthy women and found that the urethral and midstream microbiota were similar, with Lactobacillus predominating in all samples [21].

The female urinary microbiome is dominated by Lactobacillus species; particularly in healthy states, these organisms are more likely to serve a protective role. Other genera, such as Gardnerella, Corynebacterium, and Streptococcus, are also commonly present and may be context-dependent. Increased presence of anaerobes like Prevotella and Atopobiom is associated with microbial imbalance or recurrent infections, conditions which may lead to urothelial cancers [22].

### 3.3. The Effects of Bladder Cancer on Urinary Microbiome

A study conducted in 2019 by Hai Bi et al. was designed to compare the UB of patients with bladder cancer (BC) to healthy controls [23]. All samples were collected prior to antibiotic treatment. The analysis was performed using amplicon-based next-generation sequencing (NGS). The BC group expressed a higher abundance of Actinomyces species than controls. Actinomyces is a genus of Gram-positive bacteria that are either facultative or strict anaerobes, commonly found as part of the normal commensal flora of the oral cavity, gastrointestinal tract, genitourinary tract, and skin. Although typically harmless, they can become opportunistic pathogens under certain conditions. Based on the results, they concluded that Actinomyces may serve as a potential urinary biomarker for BC detection. They also observed that BC patients exhibited lower microbial diversity, suggesting that dysbiosis may be a consequence of the neoplastic process, which could contribute to disease progression, microbiome imbalances influencing the pathophysiology of BC [24].

Challenging the paradigm that urine is a sterile fluid, Oresta et al. revealed distinct UB composition in male patients with BC compared with healthy controls [25]. The key findings of their study were the increased abundance of Veillonella and Corynebacterium alongside a decreased level of Ruminococcus in the BC group. These findings underscore the immune and metabolic shifts in the organism associated with malignancy. The authors emphasized that targeting dysbiosis modulation could represent a novel strategy in the management and prevention of BC, reducing the public health impact of this pathology.

In 2023, Bukavina et al. conducted a global meta-analysis that included 125 studies [26]. Consistent evidence supporting the presence of bacterial taxa in the urine of patients with bladder cancer (BC). Their study highlighted that demographic factors and catheter-related contamination influence UB in a significant manner. Furthermore, they observed a higher abundance of certain bacterial genera, including Enterococcus, Acinetobacter, Micrococcus, and Ralstonia, in patients with BC compared with healthy individuals.

Recent evidence underscores the potential anti-inflammatory and antimicrobial properties of certain types of microbes associated with a healthier bladder environment. Parra-Grande et al. observed that female patients with higher bacterial abundance had a lower incidence of BC, supporting the hypothesis that a diverse microbiota may exert a protective effect in this pathology [27]. The tumor group evaluated in the same study showed an abundance of Enterococcus (higher in low-graded tumors), Barnesiella, Parabacteroides, Prevotella, and Lachnospiraceae, supporting the idea that potential pathogenic bacterial genera may have pathogenic potential for bladder cancer. The study reinforces sex-based differences in bladder cancer epidemiology, but it can also be a step forward for the potential use of UB as a biomarker for BC.

In addition to UB composition, Mansour et al. demonstrated elevated levels of human beta-defensins 2 and 3 (HBD2/HBD3) in the urine of BC patients compared with a healthy control group [28]. The levels of HBD were associated with altered bacterial growth in the BC cohort; more specifically, higher levels of HBD were correlated with a lower abundance of Gram-negative bacteria. Authors proposed that this pathophysiological mechanism is relevant in the progression and development of BC.

Sun JX et al. investigated the differences in UB between NMIBC and MIBC patients. In their study, patients with prior urinary catheterization, urinary tract infections (UTI), recent antibiotic therapy, and other urological pathologies were excluded [29]. They harvested tissue samples via TURBT or radical cystectomy (RC), performed by the same surgeon to ensure consistency. Furthermore, they evaluated the samples via Genomic DNA extraction using TIANamp Micro DNA Kid, followed by 16S rRNA analysis. From a total of 527 bacterial species, only 165 were commonly shared between the NMIBC and MIBC groups. The most interesting finding was that MIBC samples exhibited 265 species exclusively, with taxa genera being in low abundance in this group. Using Chao1, Shannon, and Simpson indices, microbial diversity was significantly higher in the NMIBC patient group, with a higher abundance of Firmicutes, Bacteroides, Staphylococcus, and Acinetobacter. The authors’ findings demonstrate a shift in UB diversity and composition between the two main stages of BC, suggesting that dysbiosis can be associated with tumor progression from NMIBC to muscle-invasive disease.

Biomarkers continue to play a critical role in the pathophysiology of cancers. Yuhang Z et al. identified Eubacterium sp. CAG:581 as a potential factor involved in NMIBC progression [30]. This bacterial genus was significantly more abundant in an NMIBC group compared with healthy controls.

In vitro experiments using NMIBC-derived cells were exposed to Eubacterium sp. CAG:581 showed tumor cell growth. The mechanism was mediated through ECM1 and MMP9 activation, genes known for cancer invasiveness and aggressiveness. Furthermore, a higher abundance of Eubacterium was associated with poor disease prognosis, demonstrating promising diagnostic accuracy (area under the curve-AUC of 0.79). Alongside Eubacterium, four other bacterial genera were significantly more abundant in the NMIBC group. Among these, Acinetobacter expressed the highest abundance, suggesting the potential role in tumor pathogenesis [31]. Chunxiao Chen et al. made one of the earliest efforts to map the bladder microbiome to immune checkpoint regulation for NMIBC patients [32]. They explored the correlation between UB and PD-L1 expression in 28 male NMIBC patients using 16S rRNA sequencing of tumor tissue after TURBT.

Furthermore, they divided the patients into two groups: PD-L1-positive and PD-L1-negative. The first group showed a higher abundance of bacterial genera such as: Leptotrichia, Roseomonas and Propionibacterium in a higher abundance. The second group (PD-L1 negative) showed significantly higher levels of Bacteroidetes (*p* = 0.017), Bacteroidia (*p* = 0.022), Bacteroidales (*p* = 0.025), Prevotellaceae (*p* = 0.028), and Prevotella (*p* = 0.04). This study suggests that PD-L1 expression, with an impact on immunotherapy response, is linked or associated with dysbiosis and UB.

### 3.4. The Impact of TURBT on Urinary Microbiome

Although there are multiple methods to collect urine samples, the results may vary due to possible benign comorbidities. Suchira et al. investigated a group of patients with superficial NMIBC undergoing regular cystoscopic surveillance [33]. The study compared midstream voided urine with urine obtained during cystoscopy. No significant differences between the two methods were found (alpha/beta diversity or taxa richness). The study suggested that midstream urine may be a reliable non-invasive alternative for UB assessment in the BC population.

Research suggests that the urobiome exists in healthy individuals, and imbalances in this environment may be linked to certain diseases. A study comprising 62 male patients (51 with NMIBC, 11 with MIBC, and 19 non-neoplastic controls) was performed to establish this fact. The final cohort included 40 NMIBC undergoing TURBT, midstream urine collected prior to cystoscopy, and 16S rRNA sequencing via the Illumina Miseq platform performed afterwards. Over a 12-month follow-up, 5 out of 40 NMIBC patients experienced tumor recurrence, but no progression to MIBC was noted. The most abundant bacterial classes in this group were Bacilli, Gammaproteobacteria, Actinobacteria, Bacteroidia, and Clostridia [34]. Furthermore, to improve diagnostic accuracy, Mansour et al. compared microbiota in tumor tissue and urine samples of the same patients diagnosed with NMIBC [35]. Tumor tissue consistently presented the following genera: Akkermansia, Bacteroides, Clostridium, Enterobacter, and Klebsiella, and the authors concluded that tumor-specific microbial signatures are more reliable than urine-derived samples.

For the same purpose, Fei Liu et al. investigated bladder tissue microbiome obtained from TURBT exactly from the tumor site and adjacent healthy tissue (5 mm away from the tumor margin) in the same patients [36]. After the resection, 16S rRNA sequencing was performed with the following results: Proteobacteria were the dominant phylum (54.1%) present in both healthy and tumor tissue. Using the Shannon index, alpha diversity observed a lower presence in tumor tissues, concluding a lower bacterial richness and diversity in cancerous cells. Weighted UniFrac distance (ADONIS, *p* < 0.001) revealed a statistically significant difference between tumor and healthy tissue regarding microbial richness

A recent study published in 2024 by Slusarczyk et al. aimed to examine the changes in UB for 11 patients with low-grade NMIBC [37]. They analyzed a urine sample and tumor tissue collected via TURBT, and one more urine sample after one year, as a follow-up. The researchers observed that UB decreased over the follow-up year after TURBT. Acinetobacter and Spingomonas were two species with the highest abundance in the tumor tissue samples. The study suggests that UB continues to change after tumor removal, and tumor tissue microbiomes are distinct compared with urine microbiomes.

C. Nardelli et al. designed a study to compare the UB in patients with NMIBC undergoing TURBT [38]. They analyzed first-morning (FM) urine from multiple groups: BC, benign bladder tumors, prostate cancer, and healthy controls. Alpha-diversity showed no major differences, while beta-diversity revealed significant differences in microbial composition between the BC group and control. Porphyromonas, particularly Porphyromonas somerae, was significantly more abundant in BC patients over 50 years old, compared with same-age controls, strengthening the potential role of UB in BC detection and risk stratification.

Another interesting finding is the presence of HPV and other viruses in human urine and their potential association with BC. Hrbacek et al. collected urine samples from patients undergoing TURBT and analyzed them for viral DNA [39]. Species like human cytomegalovirus, Epstein–Barr virus, Human herpesvirus-6, human papillomavirus (HPV), BK polyomavirus, torque teno virus and JC polyomavirus were found. In the BC group, HPV was found more frequently compared with control, commonly found in patients with advanced-stage disease. The association between HPV viruria and BC remains to be further explored, especially in individuals with advanced stage disease.

### 3.5. The Impact of BCG Chemotherapy on Urinary Microbiome

Intravesical Chemotherapy with BCG may be indicated in certain stages of NMIBC to improve overall survival and to reduce recurrence and progression rates of this disease. As stated before, UB can play a crucial role in response to adjuvant treatment of BC. In 2023, C. James et al. investigated the effects of intravesical therapy (IVT) on the urinary microbiome for 29 high-grade NMIBC undergoing IVT with BCG or Gemcitabine [40]. They collected urine samples before and after each treatment cycle, profiling the urobiome using 16S rRNA gene sequencing. After analyzing all data, they observed that IVT significantly reduced bacterial richness, although the diversity remained the same. Patients with recurrence of BC had a higher abundance of Aerococcus, and those without showed increased levels of Escherichia or Shigella species. The same study states that Ureaplasma levels may be expressed by a positive response to treatment and reduced incidence. In light of the findings, they concluded that Aerococcus may be associated with poor IVT response, while Escherichia, Shigella, and Ureaplasma may aid in a good therapeutic response.

### 3.6. The Impact of Toxic Substances on Urinary Microbiome

Given the high incidence of BC in smoking patients, we aimed to find more studies about the correlation between smoking and the urinary microbiome. Studies presented above revealed that the UB is closely linked to BC. In light of the correlation between BC and smoking, Wenchao Ma et al. collected urine samples from 26 male smokers (11 healthy controls and 15 diagnosed with BC) [41]. After using 16S rRNA sequencing, the researchers found that Stenotrophomonas, Enterococcaceae, Enterococcus, Myroides, and Parvimonas were higher in the BC group. Also, smokers showed greater species richness and beta-diversity compared to controls; 30 unique clusters of orthologous groups of proteins (COGs) were identified in the smoking group. The authors concluded that five bacterial taxa were enriched in the smoker group: Clostridiales, Clostridia, Bacteroidales, Bacteroidia, and Bacteroidetes.

One of the most common symptoms of BC is hematuria. The presence of it will result in an emergency urological evaluation. Due to the high numbers of BC patients diagnosed because of this symptom, Moynihan M. et al. assessed the impact of hematuria and tobacco smoking in newly diagnosed BC patients [42]. The study group comprised 43 men newly diagnosed with bladder cancer, 17 of them being current or former smokers. Urine samples were collected from midstream and analyzed via 16S rRNA sequencing. The study did not find any significant differences in alpha or beta diversity by smoking status

Another toxin that can influence the incidence of BC is arsenic, and exposure to it. Due to this well-established environmental risk factor, X. Chen et al. investigated its effects on both UB and bladder lesions in rat models [43]. The models were exposed to low or high concentrations of inorganic arsenic (iAs) via contaminated drinking water. High-dose group received 100 mg/L sodium arsenite (NaAsO2) and was reported to have an increased severity of bladder lesions. While UB did not differ between the two groups, in the high-dose group, the authors observed several urinary metabolites present: acetylornithine, prostaglandin B1, deoxyinosine, biopterin, and 1-methyluric acid. This study concluded that arsenic exposure alters urinary metabolism, but microbial diversity remains almost the same; further long-term follow-up is needed in order to assess the UB shift in this group.

Currently, there is no conclusive evidence regarding the impact of other specific toxic substances—aside from smoking and arsenic exposure—on the urinary microbiome. However, the influence of several environmental pollutants on the gut microbiota has been well documented. Notably, substances such as bisphenols, phthalates, polycyclic aromatic hydrocarbons (PAHs), heavy metals (including mercury and cadmium), and various pesticides have been shown to alter gut microbial composition and function [44]. This is of particular interest considering that gut dysbiosis, reduced concentrations of butyric acid, and impaired intestinal barrier integrity have been observed in patients with bladder cancer [45].

## 4. Discussion

This narrative review highlights the growing evidence linking the urinary microbiome to bladder cancer development in terms of progression, recurrence, and treatment response.

Recent evidence suggests that bladder cancer is associated with significant alterations in the urinary microbiome, characterized by distinct shifts in microbial composition. Species as Porphyromonas, Actinomyces, Aerococcus, and Escherichia/Shigella may be potential biomarkers of this pathology. These findings reinforce the hypothesis that a dysbiotic urinary microbiome may play a contributory role in tumorigenesis, potentially through inflammation-mediated mechanisms [46]. Moreover, additional evidence suggests that interactions between the host, environment, and tumor microenvironment may influence the composition and diversity of urinary bacterial communities [47].

Despite these suggestive findings, our understanding remains far from complete, and several points are still contested. While some studies have reported increased microbial diversity in bladder cancer patients [48], others have observed decreased or unchanged diversity, with conflicting associations between diversity levels and clinical outcomes [49,50]. These discrepancies may reflect methodological differences and population heterogeneity, and no consistent microbial signature has been identified across studies. As such, the existence of a definitive “bladder cancer microbiome” remains controversial, suggesting that dysbiosis may be context-dependent and vary across cohorts.

Another unresolved question is whether microbiome changes precede tumor development or result from it [51]. It is plausible that an underlying shift in the urinary microbiota—due to factors like long-term antibiotic use, chronic infection, or environmental exposures—could create pro-tumorigenic conditions that increase bladder cancer risk. Equally plausible is the reverse: the presence of a tumor (or associated factors like tissue necrosis, immune response, or therapeutic interventions) might itself alter the local microbial community. Currently, the temporal relationship is not definitively established, as most studies have been cross-sectional. This leads to a classic “chicken-and-egg” conundrum in bladder cancer microbiome research that future longitudinal studies will need to clarify.

Another intriguing aspect under investigation is the role of the urinary microbiome in the marked sex disparity of bladder cancer incidence. Healthy women’s urine is frequently dominated by Lactobacillus species, especially in premenopausal age, whereas men (and postmenopausal women) tend to have more diverse and anaerobe-rich urinary microbiota [52]. The presence of Lactobacillus in the bladder or vaginal environment may partially explain the lower risk of bladder cancer in women, as it likely functions as a protective commensal by inhibiting the growth of pathogenic bacteria and limiting excessive inflammation [53].

Beyond carcinogenesis, the urinary microbiome may also impact how patients respond to bladder cancer treatments. The clearest example is with intravesical Bacillus Calmette–Guérin (BCG) immunotherapy for NMIBC. Variability in patient responses to BCG has been partially attributed to differences in urinary microbiota, with some commensal microbes potentially competing with BCG for binding sites or modulating the host immune response. For instance, species like Lactobacillus iners enhance BCG’s therapeutic efficacy depending on their interactions with urothelial receptors. Clinical studies have identified correlations between specific microbial profiles and BCG treatment success: responders tend to have microbiota enriched in Firmicutes, such as Lactobacillales, whereas non-responders often harbor higher levels of Proteobacteria. These findings suggest the urinary microbiome could serve as a predictive biomarker for BCG responsiveness, and manipulating microbial composition through probiotics or other interventions may enhance therapeutic efficacy [54].

Despite growing interest in the urinary microbiome’s role in bladder cancer, current evidence is limited by substantial methodological heterogeneity and a lack of standardization across studies. Most of the studies are cross-sectional with small sample sizes and exhibit variability in sample collection and sequencing platforms, creating uncertainties regarding comparability and reproducibility. The majority of studies lack stratification by sex, tumor stage, comorbidities, and lifestyle factors, which may result in differing associations. The most significant limitation is the absence of longitudinal studies to determine a causal relationship between bladder cancer (BC) and urinary microbiome shifts. Larger prospective cohort studies with well-established protocols are essential to validate the findings presented in this study. In addition, the impact of probiotics, antibiotics, and immunotherapy in BC patients and their effect on the urinary microbiome represents a promising area of study in the future. Last but not least, a deeper understanding of the urinary microbiome’s role in bladder cancer incidence, recurrence, and prognosis could lead to the development of precise tools that can improve patient outcomes and reduce the public health burden of this pathology.

Future research on the urinary microbiome and bladder cancer should focus on standardizing methodologies for sample collection, sequencing, and bioinformatics to improve comparability across studies. Longitudinal and mechanistic studies are mandatory for establishing causality, with animal models and in vitro systems offering valuable tools for functional investigations. Prospective trials aimed to study the relationship between urinary microbiome and bladder cancer, including probiotics or microbial modulation therapies, and their effect are essential to validate the therapeutic potential.

Therapeutic modulation of the urinary microbiome, including probiotics, targeted antibiotics, or microbiota-based adjuvants, represents a promising avenue for improving treatment responses, particularly in BCG therapy. Expanding beyond bacterial analysis to include fungi and viruses may uncover additional contributors to bladder carcinogenesis and microbial imbalance. Ultimately, integrating microbiome data into personalized and preventive care models could yield significant clinical and economic benefits, especially for high-risk and recurrent bladder cancer populations.

This review synthesizes current evidence to highlight key gaps and opportunities in our understanding of microbiome–host interactions in bladder cancer. By critically evaluating existing studies, it aims to inform and shape future research directions in this emerging field. The insights gathered may support the development of more accurate diagnostic approaches and individualized therapeutic strategies. Ultimately, a deeper understanding of the urinary microbiome could contribute to improved clinical outcomes for patients with bladder cancer.

## 5. Conclusions

Recent scientific evidence has revealed that the urinary microbiome plays a role in bladder cancer pathogenesis, diagnosis, and therapeutic response. Multiple studies have shown that microbial diversity and composition differ between patients with bladder cancer and healthy patients, and BC patients. Comparative analyses of tumor tissue and urine samples have identified distinct microbial signatures. Tumor-associated microbiota exhibit greater microbial diversity compared to urine samples. Given this fact, microbial communities in tissue samples may offer superior biomarker potential compared to urine samples. Therapeutic interventions, including BCG IVT and Gemcitabine, significantly alter microbial richness; the abundance of Aerococcus and Ureaplasma may correlate with treatment efficacy or resistance. Furthermore, external exposure to tobacco smoke and inorganic arsenic not only contributes to BC risk but is also associated with important changes in urinary composition and metabolomic profiles. The detection of viral genomes, especially HPV in BC patient urine, may be implicated in tumor etiology and progression. The urinary microbiome represents a promising frontier for bladder cancer research. It may be associated with prognostic and risk stratification, and also with therapeutic response to certain treatments. Future studies should be conducted to validate the urinary microbiome’s feasibility and accuracy in managing bladder cancer. A deeper understanding of the urinary microbiome in this context could enable its integration into clinical practice. Public health systems could benefit through earlier detection, reduced recurrence, and lower surveillance costs—particularly in high-risk populations such as smokers and the elderly.

## Abbreviations

The following abbreviations are used in this manuscript:

NMIBC	Non-muscle invasive bladder cancer
EAU	European Association of Urology
TURBT	Trans-urethral resection of bladder tumor
UB	Urinary microbiome
TMB	Tumor mutational burden
BPH	Benign prostatic hyperplasia
LUTS	Lower urinary tract symptoms
STD	Sexually transmitted disorders
IPSS	International Prostate Symptom Score
NGS	Next-generation sequencing
BC	Bladder cancer
HBD	Human beta-defensins
UTI	Urinary tract infections
RC	Radical cystectomy
AUC	Area under the curve
FM	First-morning urine
HPV	Human Papillomavirus
IVT	Intravesical therapy
iAs	Inorganic arsenic
